# Respiratory Toxicology of Graphene-Based Nanomaterials: A Review

**DOI:** 10.3390/toxics12010082

**Published:** 2024-01-18

**Authors:** Chunxue Kong, Junwen Chen, Ping Li, Yukang Wu, Guowei Zhang, Bimin Sang, Rui Li, Yuqin Shi, Xiuqing Cui, Ting Zhou

**Affiliations:** 1 Environmental Toxicology Laboratory, Hubei Province Key Laboratory of Occupational Hazard Identification and Control, School of Public Health, Wuhan University of Science and Technology, Wuhan 430065, China; maggiekong24@163.com (C.K.); smilezhang163@163.com (G.Z.); sangbimin@163.com (B.S.); shiyuqin@wust.edu.cn (Y.S.); 2 Department of Pulmonary and Critical Care Medicine, Xiangyang No. 1 People’s Hospital, Hubei University of Medicine, Xiangyang 441000, China; xfyycjw@163.com (J.C.); ping_li19@163.com (P.L.); 3 Department of Physical and Chemical Laboratory, The Affiliated Wuxi Center for Disease Control and Prevention of Nanjing Medical University, Wuxi Center for Disease Control and Prevention, Wuxi 214023, China; wuyukang@foxmail.com; 4 Hubei Key Laboratory of Genetic Regulation and Integrative Biology, School of Life Sciences, Central China Normal University, Wuhan 430079, China; ruili@ccnu.edu.cn; 5 Hubei Provincial Key Laboratory for Applied Toxicology, Hubei Center for Disease Control and Prevention, Wuhan 430079, China

**Keywords:** graphene, nanomaterials, respiratory toxicology, review

## Abstract

Graphene-based nanomaterials (GBNs) consist of a single or few layers of graphene sheets or modified graphene including pristine graphene, graphene nanosheets (GNS), graphene oxide (GO), reduced graphene oxide (rGO), as well as graphene modified with various functional groups or chemicals (e.g., hydroxyl, carboxyl, and polyethylene glycol), which are frequently used in industrial and biomedical applications owing to their exceptional physicochemical properties. Given the widespread production and extensive application of GBNs, they can be disseminated in a wide range of environmental mediums, such as air, water, food, and soil. GBNs can enter the human body through various routes such as inhalation, ingestion, dermal penetration, injection, and implantation in biomedical applications, and the majority of GBNs tend to accumulate in the respiratory system. GBNs inhaled and substantially deposited in the human respiratory tract may impair lung defenses and clearance, resulting in the formation of granulomas and pulmonary fibrosis. However, the specific toxicity of the respiratory system caused by different GBNs, their influencing factors, and the underlying mechanisms remain relatively scarce. This review summarizes recent advances in the exposure, metabolism, toxicity and potential mechanisms, current limitations, and future perspectives of various GBNs in the respiratory system.

## 1. Introduction

Graphene is a novel kind of nanomaterial composed of a single layer of carbon atoms, which was initially isolated from crystalline graphite in 2004 [1]. With a thickness of merely 0.335 nm, it is presently acknowledged as the thinnest two-dimensional material, serving as the fundamental substrate for sp^2^ hybridization. Surface modification, composites, and the construction of “nano-architecture” are employed to fabricate graphene-based nanomaterials (GBNs) with diverse dimensional and spatial structures. These GBNs primarily encompass pristine graphene, graphene nanosheets (GNS), graphene oxide (GO), reduced graphene oxide (rGO), as well as graphene modified with various functional groups or chemicals such as hydroxyl, carboxyl, and polyethylene glycol. Until now, GBNs have been extensively utilized in various fields, including electronics, biomedicine, pharmaceutical engineering, and tissue engineering, owing to their advantageous characteristics such as remarkable mechanical strength, exceptional electrical and thermal conductivity, high specific surface area, and valuable antimicrobial properties [2,3]. With the widespread use of GBNs, the potential for human exposure raises concerns about the safety of GBNs that showed potential behavioral, immune, pulmonary, systemic, ocular, reproductive, and developmental toxicity and genotoxicity [4,5]. Currently, reproductive, immunologic, and cytotoxicity reviews of GBNs have been conducted, but the respiratory system, as a major target of GBNs, has not yet been reported to comprehensively characterize and compare the potential impacts of different GBNs on the respiratory system. At present, toxicological studies on GBNs have focused on laboratory animal and cellular experiments, with a lack of epidemiological data on populations exposed to GBNs. Therefore, this review summarizes recent findings on the exposure routes, toxicological effects, and underlying toxicity mechanisms of GBNs in the respiratory system from in vitro and in vivo experiments.

## 2. Database Search for Articles on GBNs and Respiratory Systems 

We conducted a thorough search using well-known databases, including “Web of Science” and “PubMed” databases, using relevant keywords such as graphene, respiratory system, lung, pulmonary, laboratory experiments, and toxicity mechanism. Additionally, another search was performed with keywords including nanomaterials, pulmonary, and mechanism. The study merged and combined research results obtained from all search engines. Afterward, GBN-related results were combined with laboratory experiments on the respiratory system to enhance the study. 

Inclusion criteria were (1) in vivo and in vitro studies on graphene, GNS, GO, rGO, and functionalized and/or composite forms of graphene; (2) monitoring data in the work environment; (3) English language. The article systematically excluded information on other nanomaterials, such as carbon nanotubes, modeling methods, practical applications, and systemic toxicity beyond the respiratory system. The selection of articles has no time limit. 

## 3. Exposure and Metabolism of GBNs

GBNs are known to exhibit high resistance to degradation once introduced into the natural environment, facilitating their transfer or enrichment among environmental mediators or organisms, with direct or indirect toxic effects on both aquatic and terrestrial animals and plants [6]. Several investigations have indicated that GBNs can infiltrate organisms via various routes such as skin, respiratory tract, digestive tract, and eyes, subsequently accumulating in tissues and organs such as the lung, liver, spleen, and kidney [7,8]. Although human data, case reports, or medical investigations of workers exposed to GBNs are not currently available, monitoring data in the work environment and laboratory data on GBN toxicity are important for the health risk assessment of GBNs.

At present, only a few works of literature have reported airborne GBN concentrations in occupational environments. Graphene concentrations are mostly below detection limits in workplace monitoring and in studies modeling graphene exposure [9,10]. In a large-scale graphene production workplace, occupational exposure to GBNs by workers was found to be between 909 and 6438 particles per cm^3^ after an average exposure of 8 h, which is equivalent to 0.38–3.86 μg·cm^−3^ [11]. Airborne GNS concentrations of 2.27 and 0.017 mg·m^−3^ were measured when collecting the products from the discharge vessel [12]. This concentration range is similar to the range that did not cause toxicity in rats during a five-day repeated exposure to nasal inhalation only of GNS (0.12–1.88 mg·m^−3^) [13]. Although airborne levels of GBNs in the occupational environment seem to be lower, it is noteworthy that the chronic toxicity and/or carcinogenicity of various GBNs should not be underestimated.

Animal experiments have demonstrated that the elimination of GBNs from the body is primarily contingent upon their size, with smaller GBNs being rapidly removed, while larger GBNs are more prone to retention [14]. Specifically, the larger GBNs entering via the respiratory tract tended to persist in the non-ciliated region for a long time, leading to the development of lung inflammation, edema, granuloma, and fibrosis [15]. In addition, the surface of GBNs can be modified through polyethylene glycol (PEG) functionalization, resulting in a reduction in lung inflammatory and toxic responses caused by GBNs [16]. This modification has been found to have positive biological effects, including antioxidant, anti-apoptotic, antibacterial, and anticancer effects [17]. Therefore, a comprehensive understanding of the effects of GBNs on the respiratory system is more conducive to their application and development in various fields. 

## 4. Respiratory Toxicity of GBNs

**Figure 1:**
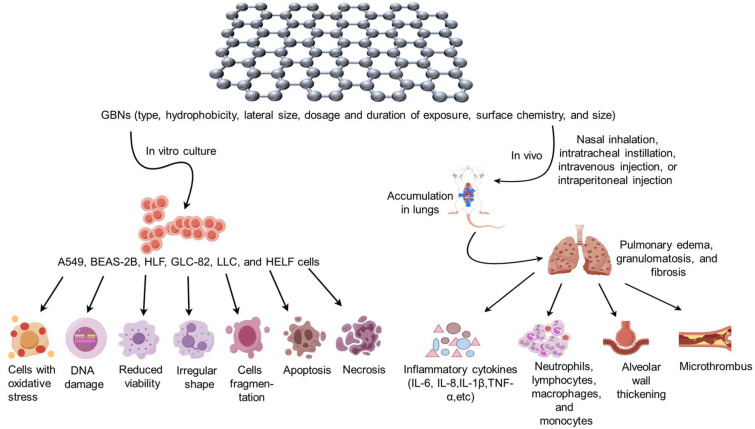
(By Figdraw). Respiratory toxicity induced by graphene-based nanomaterial (GBN) exposure in vitro and in vivo. When cultured in vitro with the addition of GBNs, lung cancer human alveolar basal epithelial cells (A549), human bronchial epithelial cells (BEAS-2B), human lung fibroblast cells (HLF), lung cancer cell line (GLC-82), Lewis lung carcinoma (LLC), and human embryonic lung fibroblasts (HELF) exhibited oxidative stress, DNA damage, decreased viability, irregular cell shape, cell fragmentation, apoptosis, necrosis, and other related phenomena. In the in vivo experiments, various types, sizes, dosages, and duration of exposure of GBNs were administered to the animal organism through nasal inhalation, intratracheal instillation, intravenous injection, intraperitoneal injection, etc. This resulted in the accumulation of GBNs in the lungs, causing pathological changes such as pulmonary edema, granulomatosis, and fibrosis. The study observed an increase in the expression of inflammatory cytokines, including IL-6, IL-8, IL-1β, and TNF-α, as well as an increase in the number of inflammatory cells such as neutrophils and lymphocytes. Additionally, thickening of alveolar walls and formation of microthrombus were observed.

Animal models of GBN toxicity can be constructed by diverse exposure routes, including nasal inhalation, intratracheal instillation, intravenous injection, and intraperitoneal injection, leading to the development of respiratory disorders such as lung inflammation, fibrosis, and granuloma. Furthermore, the surface modification of GBNs with various functional groups can potentially exhibit both beneficial biological effects, such as anti-inflammatory and anticancer properties, as well as exacerbate the toxic effects associated with GBNs. Consequently, this review paper aims to elucidate the respiratory toxicity characteristics of different types of GBNs, discern their distinctions, and establish a more comprehensive foundation for toxicity assessment in the extensive utilization of GBNs. And the respiratory toxicity induced by GBN exposure in vitro and in vivo is summarized in Figure 1 and Table 1.

### 4.1. Graphene and GNS

In vitro cellular experiments revealed that human bronchial epithelial cells (BEAS-2B) or lung cancer human alveolar basal epithelial cells (A549) exposed to graphene or GNS showed a notable reduction in cell viability, accompanied by the occurrence of apoptosis and necrosis in a dose- and time-dependent manner [18,19,20]. Animal studies indicated that, following nasal inhalation of graphene at concentrations ranging from 0.68 to 3.86 mg/m^3^ for 6 h per day, there was a slight increase in the number of neutrophils, lymphocytes, and monocytes in the peripheral blood on day 1, but the count of white blood cells and lymphocytes returned to a normal level by day 3. Conversely, no discernible pathological alterations such as inflammation, fibrosis, or granuloma formation were observed in the lungs of mice following a single intratracheal administration of 50 μg of graphene on days 1, 7, and 42. And well-dispersed graphene would induce more minimal toxicity in the lung than aggregated graphene [21,22]. At present, the toxicity of graphene has not been fully elucidated, so its potential toxicity is still not to be underestimated and deserves our attention.

On days 1, 28, and 90, nasal inhalation of GNS at concentrations ranging from 0.1 to 3.2 mg/m^3^ (lateral size < 2 μm or 2–7 μm) for 6 h per day, five days per week, did not result in any pulmonary toxicity or proinflammatory effects in rats [13,23]. However, when a single intratracheal instillation of GNS (lateral size < 2 μm) at a dose from 0.3 to 3 mg was administered on day 1, an increase in the number of neutrophils in the bronchoalveolar lavage fluid (BALF) of the rats was observed in a dose-dependent relationship, and acute multifocal alveolitis occurred, which disappeared from the lungs of the rats on day 28 [24]. But in mice, a single intratracheal administration of fewer layers (1–10 layers) of 50 μg GNS with a lateral size less than 4 μm that were phagocytosed by macrophages remained in the lungs and metastasized to the mediastinal lymph nodes, inducing high levels of inflammatory cytokines, including IL-1β, IL-6, TNF-α, MIP-2, and IL-8 in BALF during the period of 1–7 days following [25]. Additionally, the lungs of mice exhibited pathological changes such as inflammatory edema and granulomas, which were predominantly infiltrated by neutrophils and eosinophils. However, with the prolongation of time, the lung structure of the mice gradually returned to normal by the 28th day after exposure to GNS [18,25,26]. Furthermore, 4 h after a single intratracheal administration of 40 μg GNS with lateral sizes smaller than 2 μm, 5 μm, and 20 μm, the levels of neutrophil counts, macrophage colony-stimulating factor (MCSF), myeloperoxidase (MPO), and lactate dehydrogenase (LDH) expression in BALF were significantly elevated for GNS with lateral sizes of 5 μm and 20 μm compared to those with a lateral size smaller than 2 μm. And the inflammation persisted in the lungs on day 30, returning to normal on day 60 [27]. However, the research has demonstrated that even on day 90 after a single intratracheal instillation of 25–100 μg of GNS (with a lateral dimension of approximately 500 nm), GNS remains present in the lung tissue of mice and induces alterations in the lung gene profile and the expression level of Th1/Th2 cytokines, which subsequently disrupt the physiological and immune homeostasis of the organism [28]. Consequently, it can be concluded that GNS exerts a certain degree of toxicity on lung cells. However, it is important to note that the toxic effects on the lungs may vary depending on the distinct physical properties of GNS such as lay number, lateral size, exposure route, and dose, as well as animal species. When GNS with a high concentration or transverse size enters the lungs, it can induce acute inflammatory damage. However, with the prolongation of time, the lungs possess the capacity for self-repair, resulting in gradual restoration of the inflammatory lesions to their normal state. Nevertheless, due to the diminutive size of GNSs, it is difficult to eliminate them from the body, so they could accumulate in the lungs for a long time and readily traverse the gas–blood barrier during entry into the circulatory system, thereby causing an imbalance in lung immunity and the occurrence of chronic inflammation.

### 4.2. GO

In vitro cellular assays have demonstrated that GO exhibits dose- or time-dependent cytotoxicity and genotoxicity against a variety of cell types, including A549, BEAS-2B, human bronchial epithelial cells (16HBE), human lung fibroblast (HLF) cells, and a lung cancer cell line (GLC-82). The toxicity of GO derived from different oxidation methods exhibits variability in A549 cells, with toxicity increasing as the oxygen content rises [29]. At concentrations ranging from 1 to 100 μg/mL, GO shows a significant decrease in the viability of HLF cells, accompanied by an increase in intracellular ROS production and the occurrence of cellular morphological irregularities, fragmentation, or apoptosis [30]. But there was no significant cytotoxicity in the murine lung epithelial cell line (FE1) after exposure to GO mainly consisting of 2–3 graphene layers with a lateral size of 1–2 μm at relatively high doses (5–200 μg/mL), even if excessive ROS were generated [31].

In animal experiments, no obvious pathological alterations were observed in the lungs of rats following active nasal intranasal inhalation of 0.6–10 mg/m^3^ GO (0.5–5 μm lateral size) for a duration of 5 days [32]. However, exposure to 0.05–500 mg/kg GO (0.1–10 μm) or nanographene oxide (NGO) by intratracheal instillation, tail intravenous injection, or intraperitoneal injection could lead to acute and chronic pulmonary inflammatory damage with progressive dose-dependent aggravation in mice. There were obvious pathological changes in the lungs, mainly characterized by neutrophil infiltration, increased pulmonary capillary permeability, microthrombosis, alveolar wall thickening, epithelioid granulomas, pulmonary edema, and peribronchiolar collagen deposition on day 7 after exposure. And elevated levels of LDH, alkaline phosphatase (ALP), TNF-α, IL-6, and IL-1β were also shown in a marked dose-dependent manner [16,33,34,35,36,37,38,39]. On day 90, it was observed that NGO had not been completely eliminated from the lungs [40]. Furthermore, the size of GO plays a significant role in its toxicity [41], as micro-sized GO particles exhibit a reduced clearance rate in the lungs and induce more severe inflammation and fibrosis compared to nano-sized GO particles [42,43,44,45]. 

In an animal model of ovalbumin (OVA)-induced allergic asthma, administration of GO via daily intraperitoneal injection at doses ranging from 0.04 to 4 mg/kg during the sensitization period resulted in a deterioration of airway hyperresponsiveness and collagen deposition, elevated IL-4, and decreased IFN-γ expression in the lungs on day 40 [46], indicating marked exacerbation of OVA-induced asthmatic responses, as the dose of GO was increased. Another study discovered that a single intratracheal administration of 80 μg of GO on the first day of OVA sensitization significantly reduced eosinophil accumulation and attenuated Th2 immune response in the lung on the second day following challenge (31 days after sensitization). However, this intervention also led to an increase in the level of acidic mammalian chitinase (AMCase) released by alveolar macrophages, as well as heightened airway hyperresponsiveness and airway remodeling [47]. 

Therefore, it can be postulated that the impact of GO on the induction of lung damage in animals is influenced by various factors, including size and oxygen content of GO, concentration of the administered dose, duration of exposure, pathways into the body, as well as characteristics of animal species, exogenous toxicant, and underlying disease.

### 4.3. rGO

Currently, the primary focus of in vitro cellular experiments involving rGO is the examination of the effects on FE1 and A549 cells. rGO with a lateral size of 1–2 µm did not induce significant cytotoxicity or genotoxicity in FE1 cells at relatively high concentrations (5–200 µg/mL) [31]. However, rGO with a lateral size from 50 to 700 nm enhanced the migration and invasion of A549 cells at concentrations ranging from 1 to 10 µg/mL, whereas rGO at a concentration of 20 µg/mL suppressed cell migration and invasion without affecting cell viability [48]. However, it was also discovered that concentrations equal to or greater than 5 μg/mL of rGO, with a lateral size of approximately 900 nm, significantly inhibited A549 cell viability in a dose-dependent manner. This inhibitory effect was accompanied by an increase in intracellular ROS production, activation of the NF-κB pathway, release of IL-8 expression, and induction of apoptosis [3,49,50]. The variation in cytotoxicity primarily depends on the lateral size of the rGO, whereby larger sizes have a more pronounced impact on cell viability at equivalent concentrations. Another animal experiment conducted intratracheal instillation administration of 18–162 μg of rGO (1–2 μm), which resulted in a predominantly neutrophilic inflammatory response in the lungs of mice from day 1 to day 90, with increased DNA damage in the inflammatory cells of BALF in a clear dose-effect relationship; however, no fibrosis formation was observed [9].

### 4.4. Functionalized GBNs

Functionalized GBNs are a subset of GBNs that are distinguished by their integration of functional groups (e.g., hydroxyl groups, epoxy groups, etc.), which are formed during the preparation process. These functional groups are attached to the graphene surface through covalent bonding, noncovalent interactions, or doping [51]. Consequently, functionalized GBNs possess a combination of properties derived from both graphene and the modified functional groups. There are two main categories into which they can be broadly classified: graphene/inorganic nanocomposites, which encompass metals and carbon nanomodified materials, and graphene/polymer composites, which involve synthetic polymers and modifications of natural polymers. Functionalized GBNs exhibit anticancer, anti-inflammatory, and anti-apoptotic properties, thereby reducing the toxicity of GBNs and enhancing their potential as drug adjuvants. Nevertheless, it should be acknowledged that the vanillin modification could exacerbate the toxic effects of GBNs [52].

#### 4.4.1. GBN/Inorganic Nanocomposites

Respiratory toxicity studies of GBN/inorganic nanocomposites were primarily conducted on lung fibroblasts and lung cancer cells, specifically human embryonic lung fibroblasts (HELF), A549 cells, human large cell lung cancer (H460), human non-small-cell lung cancer (H1975), and Lewis lung carcinoma (LLC). According to recent studies [53,54], incubation of HELF, A549, H460, H1975, and LLC cells with graphene/nickel oxide nanocomposites (Gr/NiO NCs) and iron/platinum nanoparticle GO (FePt/GO) at concentrations ranging from 0 to 100 μg/mL for 24 h did not yield any significant alterations in the cell viability of HELF, but GBN/inorganic nanocomposites decrease other cancer cell viability and cell death rate in a dose-dependent manner, indicating an enhanced anticancer efficacy. To serve as a drug delivery method, composite materials consisting of synthesized graphene oxide/TiO_2_/doxorubicin (GO/TiO_2_/DOX) were incorporated into a chitosan/poly (lactic acid) (PLA) solution. The increased concentrations of GO/TiO_2_/DOX significantly suppressed the proliferative capacity of A549 lung cancer cells [55].

#### 4.4.2. GBN/Polymer Composites

In the cellular experiments, it was observed that amino-modified GNSs effectively reduced inflammation and genotoxicity induced by GNSs in human-transformed type I (TT1) alveolar epithelial cells and human lung epithelial cells (16HBE14o-) [56,57,58]. On the other hand, at concentrations ranging from 1 to 100 μg/mL, PEG-modified GO showed significantly lower toxicity towards HLF cells compared to unmodified GO. In contrast, GO modified with polyethylenimine (PEI) exhibited a notable increase in its cytotoxic effect [30].

Animal experiments have demonstrated that the levels of LDH and total protein in the BALF of rats were elevated and accompanied by a predominantly neutrophilic inflammation on day 1 following the intratracheal instillation of GNS (1 mg/rat) modified with carboxy (COOH), N–H, and N=H, respectively, but this inflammation gradually diminished over a period of 28 days [24]. Additionally, after three months of administration of poly sodium 4-styrenesulfonate (PSS) within the dosage range of 0–16 mg/kg or PEG-modified GO at a dosage of 5 mg/kg via tail intravenous injection, the lungs of mice exhibited a dose-dependent chronic inflammation with macrophage infiltration, whereas PEG modification accelerated GO clearance, attenuated collagen deposition in lung tissue, and induced chronic lung fibrosis [59,60]. It can be hypothesized that the toxicity and subsequent biological effects of GBN/polymer composites primarily rely on the physicochemical characteristics of the surface-modified complexes, which determine their ability to remove GBNs and alleviate their toxicity and inflammatory responses. Consequently, these properties dominate the potential and applicability of these composites in the biomedical field.

GBN/polymer composites are mainly utilized to exploit the superior biocompatibility and hydrophilicity of GBNs, as well as other physical properties, to serve as carriers to enhance the hydrophobicity of cancer therapeutics, thereby improving the targeting of cancer therapies to cancer cells. For instance, it has been observed that a concentration of 1 μg/mL of doxorubicin (DOX)-loaded hyaluronic acid (HA)-modified Q- graphene is capable of inducing a 50% reduction in A549 cells [61]. Similarly, the inhibitory effect of anticancer drugs on the growth and proliferation of A549 cells and NSCLC cell line NCI-H460 can be enhanced by the use of oridonin-loaded chitosan GO (CS-GO), HA-functionalized GO-based gefitinib delivery system (NGO-SS-HA-Gef), and paclitaxel-loaded GO (PTX-GO). These delivery systems have concentrations ranging from 16 to 64 μg/mL for CS-GO, 1 to 400 μg/mL for NGO-SS-HA-Gef, and 10 to 150 μg/mL for PTX-GO [62,63,64]. 

There was a dose-dependent decrease in cell viability and an increase in ROS production when A549 cells were exposed to varying concentrations (1–1000 µg/mL) of polyethylene glycol (PEG), polyphenol-modified rGO (PrGO), and Pulicaria glutinosa extract/silver/highly rGO (PGE/Ag/HRG). And these modified GOs induced A549 cell apoptosis with a decreased percentage of cells in the G0/G1 phase and the increased accumulation of cells in the subG1 phase, indicating a potential resistance to cancer cell proliferation [3,65,66]. It is suggested that GBNs could be utilized as efficient drug carriers for the treatment of lung cancer and exhibit anticancer properties. Despite their adverse impact on the lungs, functionalized GBNs hold promise as nanocarriers or adjuvants, thereby presenting significant potential in the clinical management of lung cancer.

**Table 1:** The toxicity of graphene-based nanomaterials (GBNs) in vitro and in vivo.

Exposure Model	Type	Lateral Size	Animals/Cells	Exposure Route	Exposure Concentration/Dose	Exposure Duration/Frequency	Time Points Post-Exposure	Toxicity	Adverse Effects	Ref.
In vitro										
	Graphene and GNS	<2 μm	BEAS-2B, A549 cell	--	0.05~1000 μg /mL	--	6, 24, 48, and 72 h and 7 days	Yes	Reduce cell viability, apoptosis	[18,19,20]
	GO	200~500 nm	HLF cell	--	1~100 μg/mL	--	2, 4, 12, and 24 h	Yes	ROS production, cellular morphological irregularity, fragmentation or apoptosis	[30]
	GO	1~2 μm	FE1 cell	--	5~200 μg/mL	--	24 h	No	--	[31]
	rGO	1~2 μm	FE1 cell	--	5~200 μg/mL	--	24 h	No	--	[31]
	rGO	50–700 nm	A549 cell	--	1~20 μg/mL	--	48 and 72 h	No	--	[48]
	rGO	approximately 900 nm	A549 cell	--	≥5 μg/mL	--	6 and 24 h	Yes	Cell viability inhibition, apoptosis	[3,49,50]
	Gr/NiO NCs, FePt/GO	4~15 nm	HELF, A549, H460, H1975 and LLC cell	--	0~100 μg/mL	--	24 h	Yes	No change in the viability of HELF cells, decrease other cancer cell viability and cell death rate	[53,54]
	GO/TiO_2_/DOX	--	A549 cell	--	50~1500 mg	--	24, 48, and 96 h	Yes	Suppress the proliferative capacity	[55]
	amino-modified GNS	10 μm	TT1, 16HBE14o-cell	--	0~100 μg/mL	--	16 and 24 h	No	Reduce inflammation and genotoxicity	[56,57,58]
	PEG-GO	50~150 nm	HLF cell	--	1~100 μg/mL	--	24 h	Yes	Lower toxicity compared to unmodified GO	[30]
	PEI-GO	200~500 nm	HLF cell	--	1~100 μg/mL	--	24 h	Yes	DNA damage of HLF cells	[30]
	DOX-HA-Q-GS	--	A549 cell	--	1 µg/mL	--	48 h	Yes	Apoptosis	[61]
	CS-GO, NGO-SS-HA-Gef, PTX-GO	50~200 nm	A549, NCI-H460 cell	--	16~64 µg/mL, 1~400 µg/mL, 10~150 µg/mL	--	12, 24, 36, 48, and 72 h	Yes	Inhibitory effect of on the growth and proliferation of cell, apoptosis	[62,63,64]
	PEG-rGO, PrGO, PGE/Ag /HRG	791.37 nm	A549 cell	--	1~1000 µg/mL	--	24 and 48 h	Yes	Decrease in cell viability, ROS production, decrease percentage of cells in the G0/G1 phase and increase accumulation of cells in the subG1 phase, apoptosis	[3,65,66]
In vivo										
	Graphene	10~130 nm	Rat	nasal inhalation	0.68~3.86 mg/m^3^	6 h/day for 5 days	1, 3, 7, and 28 days	Yes	Slight increase in the number of neutrophils, lymphocytes, and monocytes, the count of white blood cells, and lymphocytes	[22]
	Graphene	1.33~3.26 μm	Mice	intratracheal instillation	50 μg	Single administration	1, 7, and 42 days	No	--	[21]
	GNS	123 nm	Rat	nasal inhalation	0.12~1.88 mg/m^3^	6 h/day for 5 days	1, 28, and 90 days	No	--	[13]
	GNS	2~7 μm	Rat	nasal inhalation	0.2~3.2 mg/m^3^	6 h/day for 20 days	1 and 29 days	No	--	[23]
	GNS	2 μm	Rat	intratracheal instillation	0.3, 1, 3 mg	Single administration	1, 7, and 28 days	Yes	Increase the number of neutrophils, acute multifocal alveolitis	[24]
	GNS	<4 μm	Mice	intratracheal instillation	50 μg	Single administration	1 and 7 days	Yes	Inflammatory cytokines, inflammatory edema, and granulomas	[25]
	GNS	<2 μm, 5 μm, 20 μm	Mice	intratracheal instillation	40 μg	Single administration	4 h, 1, 7, 28, and 60 days	Yes	Increase the number of neutrophils, increase activity levels of MCSF, MPO, and LDH	[27]
	GNS	Approximately 500 nm	Mice	intratracheal instillation	25~100 μg/mice	Single administration	90 days	Yes	Alterations in the lung gene profile and the expression level of Th1/Th2 cytokines	[28]
	GO	0.5~5 μm	Rat	intranasal inhalation	0.60~10 mg/m^3^	6 h/day for 5 days	1, 3, and 21 days	No	--	[32]
	GO	0.1~10 μm	Mice, rat	intratracheal instillation, tail intravenous injection, intraperitoneal injection	0.05~500 mg/kg	Once a day for 7 days	2, 5, 10, 15, 30, and 180 min; 1, 7, 14, 28, 30, and 56 days	Yes	Neutrophils infiltration, increased pulmonary capillary permeability, microthrombosis, alveolar wall thickening, epithelioid granulomas, pulmonary edema, and peribronchiolar collagen deposition	[16,33,34,35,36,37,38,39]
	GO	--	Mice	intraperitoneal injection	0.04~4 mg/kg	32 days	8 days	Yes	Deterioration of airway hyperresponsiveness, collagen deposition, elevated IL-4, and decreased IFN-γ	[46]
	GO	--	Mice	intratracheal instillation	80 μg	Single administration	31 days	Yes	Promoted airway hyperresponsiveness, airway remodeling, subepithelial fibrosis reducing eosinophilic inflammation in favor of the emergence of monocytes/macrophages, reduced OVA-induced AMCase enzymatic activity in the BALF	[47]
	GO	--	Mice	intratracheal instillation	40 μg/day	Once a day for 2 days	2 days	Yes	Facilitated transient neutrophil accumulation in BALF, reduced OVA-induced AMCase enzymatic activity in the BALF	[47]
	rGO	1~2 μm	Mice	intratracheal instillation	18~162 μg/mice	Single administration	1, 3, 28, and 90 days	Yes	Neutrophils inflammatory response, DNA damage	[9]
	COOH-/N-H-/N = H -modified GNS	2 μm	Rat	intratracheal instillation	1 mg/rat	Single administration	1, 7, and 28 days	Yes	LDH and total protein elevation, neutrophilic inflammation	[24]
	PSS/PEG-modified GO	500 nm; 314 nm	Mice	tail intravenous injection	0~16 mg/kg	Single administration	1, 7, 14, 28, 30, 90, and 180 days	Yes	Chronic inflammation with macrophage infiltration	[59,60]

## 5. Mechanisms of GBN-Induced Respiratory Toxicity 

**Figure 2:**
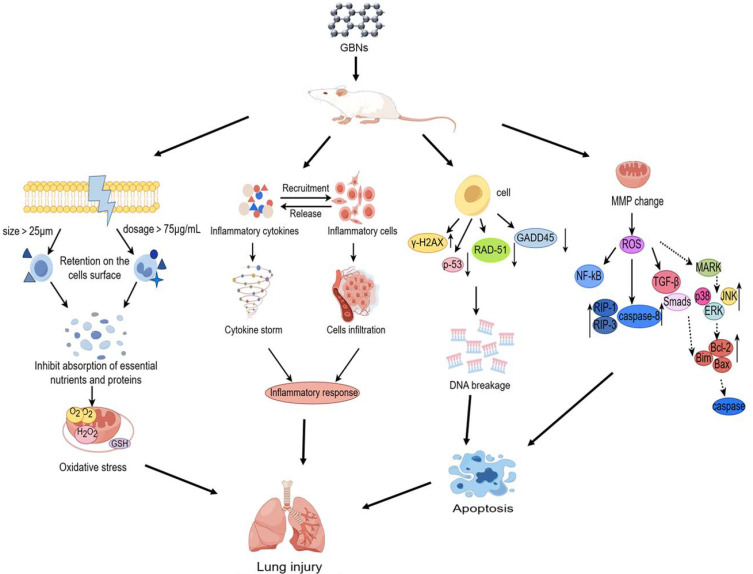
(By Figdraw). The mechanisms of GBN-induced respiratory toxicity. The toxicological mechanism of GBN-induced lung injury involves the following aspects. Oxidative stress induced by GBNs with a size greater than 25 μm or with a dose higher than 75 μg/mL adhere to the cell surface, impeding nutrient absorption and causing cellular oxidative stress. Inflammatory response induced by GBNs stimulates the release of cytokines, which can cause a cytokine storm. This can lead to the infiltration of a large number of inflammatory cells and ultimately result in lung injury. Additionally, GBNs can cause DNA damage, as evidenced by an increased expression level of the DNA breakage protein γH2AX in cells, downregulation of the expression of DNA repair proteins including p-53, RAD-51, and GADD45, and apoptosis due to DNA breaks. A change in mitochondrial membrane potential results in an increase in intracellular reactive oxygen species. This, in turn, activates several apoptosis-related signaling pathways, such as NF-κB, TGF-β/Smads, and MAPK, and leads to elevated expression of apoptotic proteins, ultimately causing apoptosis.

In terms of toxicity, a number of studies have been reported, but the field is too young and the literature is too limited for the mechanisms of uptake, interactions, and intracellular fate in the respiratory system. A recent transcriptomic analysis indicated significantly perturbed canonical pathways including pathogen recognition, immune response, cellular signaling, and cellular growth in the lung when exposed to GO or rGO [67]. However, there is a lack of research on the mechanisms of respiratory toxicity, and most studies on the specific mechanisms of respiratory toxicity of GBNs have been mainly limited to in vitro cell damage experiments related to the respiratory system, focusing on oxidative stress, inflammatory response, DNA damage, and apoptosis, as shown in Figure 2.

### 5.1. Oxidative Stress

Oxidative stress refers to an imbalance between the body’s oxidative and antioxidative mechanisms, which arises from excessive production of free radicals surpassing the capacity of antioxidant enzymes like superoxide dismutase (SOD) and glutathione (GSH) [68]. Direct or indirect generation of excessive ROS leading to oxidative stress in target cells can promote the alteration of macromolecules such as polyunsaturated fatty acids in membrane lipids, protein denaturation, DNA damage, and epigenetic alterations [69]. Previous research has indicated that GO, rGO, and CeO_2_-RGO induce oxidative stress in HLF and A549 cells by reducing GSH concentration and elevating ROS production [70,71]. However, it is not clear whether the production of oxidants is related to reactive edge sites or an indirect response of target cells to GBNs. A previous study showed that few-layer graphene with a lateral size of up to 5 μm was readily internalized by human monocytic cell line (THP-1), but the cells initially adhered to the surface and then gradually spread around and covered the surface of these large GNSs when exposed to few-layer graphene up to 25 μm [72]. Similarly, hydrophobic pristine graphene was largely retained on the cell surface of macrophages at concentrations above 75 μg/mL, which also induced oxidative stress, mainly due to strong hydrophobic interactions with the cell membrane inhibiting the uptake of essential nutrients and proteins into cells [73,74]. These suggest that dosage and physicochemical properties such as hydrophobicity, lateral size, and lay number of GBNs may be key variables in determining the source of cellular oxidative products.

### 5.2. Inflammatory Response

The inflammatory response is an extensively researched mechanism of toxicity associated with GBNs, which could upregulate the expression levels of inflammatory cytokines, triggering inflammatory cell infiltration, lung fibrosis, and granuloma formation. GO induces an elevation in the quantity of neutrophils present in BALF, accompanied by a significant increase in the expression of inflammatory factors such as TNF-α, IL-6, IL-1β, IL-8, LIX, and MCP-1 [9,34,75]. In addition, both GO and rGO exposure resulted in the activation of annotations related to mast cells and eosinophils in the hypersensitivity response function. It has demonstrated that increasing *Tnfα*, *Cxcl5*, *Saa3*, and *Ccl7* expression levels induced by GO or rGO were associated with the increased influx of neutrophils in BALF. And the expression levels of *Il1β* and *Ccl2* were found to be positively correlated with lymphocyte cells, while *Il16* exhibited a negative association with lymphocyte cell numbers in BALF. Only *Ccl24* showed a positive significant association with increased eosinophil level. The inflammatory pathways were mainly involved in granulocyte adhesion and diapedesis, IL-10 signaling, and acute phase response signaling after exposure to GO or rGO [18,27,67,76,77]. Notably, exposure to GO had a significantly more pronounced impact on the innate immune system compared to rGO exposure, activating key innate pathways such as IL-8 and HMGB1 signaling pathways, as well as IL-6 and LXR/RXR activation pathways involved in neutrophil recruitment [78,79]. In contrast, exposure to rGO specifically upregulated *Ccl5* expression, a cytokine associated with decreased neutrophil infiltration. However, rGO exposure uniquely activated several pathways involved in the activation and maturation of the adaptive immune system, such as the CD28 signaling pathway in T helper cells and the FcγRIIB signaling pathway in B lymphocytes [67]. Additionally, exposure to pristine graphene could reprogram macrophages, enhancing their response to TLR ligand stimulation by epigenetic reprogramming, specific histone methylation, and, to a lesser extent, histone demethylation [80]. Subsequent studies need to further elucidate the distinction between the roles of the respective immune cells in the innate and adaptive immune responses during exposure to different GBNs, which may contribute to the health hazard assessment of GBNs.

### 5.3. Genotoxicity and Epigenetics

Genotoxicity can be caused by gene mutations, chromosomal aberrations, or DNA damage, which poses a major threat to cell survival and can lead to cellular senescence, programmed cell death, and even tumorigenesis if intracellular DNA damage is not repaired in a timely manner [81]. Some in vivo experiments have shown that lung genotoxic effects such as DNA damage in the lung cells, chromosomal aberration in the bone marrow, and formation of micronucleated polychromic erythrocytes were exacerbated with repeated intraperitoneal or intravenous injection of GO in mice [38,82]. Graphene without or with amine and carboxyl surface functional groups showed significant micronucleus induction in TT1 alveolar epithelial cells and 16HBE14o- cells in vitro [56,57]. Five different representatives of GBNs including pristine graphene, carboxylated GNS (GNS-COOH), aminated GNS (GNS-NH_2_), single-layer GO (SLGO) and few-layer GO (FLGO) all led to single-stranded DNA damage in BEAS-2B cells, and the extent of the damage was sequentially increased at a concentration of 10 μg/mL. Although GBNs induced DNA strand breaks in A549 and HLF cells in a dose-dependent manner, acid polyethylene glycol (LA-PEG) and PEG-modified GO induced only mild DNA damage and reduced the genotoxicity of GO on HLF cells [9,30]. However, other functionalized GBNs (MFP-FePt-GO NCs) can upregulate the DNA breakage protein γ-H2AX and downregulate the DNA repair proteins such as p-53, RAD-51, and GADD45 in non-small-cell lung cancer cells (NSCLC cells), thereby enhancing DNA breakage, which in turn promotes the anticancer effects of drugs [83]. It is suggested that the potential genotoxicity of GBNs may be attributed to their impact on nucleotide excision repair and nonhomologous end-joining repair mechanisms [58]. However, it is worth noting that the magnitude of genotoxicity of modified GBNs in the respiratory system often depends on the physicochemical properties of the modified functional groups and the cell type.

Epigenetics refers to changes in gene expression levels based on non-gene sequence changes, such as DNA methylation and chromatin conformational changes. A recent study has found that medium-term exposure to rGO at concentrations of 1 and 10 μg/mL had no significant effects on the genome-wide or global DNA methylation dynamics of BEAS-2B cells [84]. However, SLGO or FLGO exposure led to a dose-dependent increase in global DNA methylation (hypermethylation) in BEAS-2B cells, and conversely, other GBN treatments resulted in cellular hypomethylation in the order of GNS-COOH > GNS-NH_2_ ≥ pristine graphene. This may be explained by DNA methyltransferase (DNMT3B gene) and methyl-CpG binding domain protein (MBD1) gene expression levels, as SLGO or FLGO upregulated their levels to promote global DNA hypermethylation while other GBNs reduced their levels to induce whole-gene hypomethylation [58]. In addition, exposure to GO at concentrations above 50 μg/mL resulted in 628 upregulated and 25 downregulated microRNAs (miRNAs), which could induce cell death and apoptosis by affecting TNF-α receptor and caspase-3, as well as the mitochondrial pathway through p53 and Bcl-2 in GLC-82 lung adenocarcinoma cells [85]. Currently, there are relatively few studies on the epigenetic toxicity of GBNs in the respiratory system, and it is still challenging to explain the mechanisms by which GBNs cause epigenetics. 

### 5.4. Apoptosis

Cell death has physiological and pathological functions; apoptosis is one type of cell death that plays a crucial role in maintaining homeostasis in normal tissues by regulating the balance between cell proliferation and cell death [86]. The induction of apoptosis by GBNs occurs through various mechanisms, including the modulation of mitochondrial membrane potential (MMP), the generation of ROS, and the signaling pathways of activated NF-κB and mitogen-activated protein kinase (MAPK), as well as transforming growth factor beta (TGF-β) [3,66,87]. rGO could lead to apoptosis in A549 cells by decreasing the MMP, upregulating apoptosis-related proteins such as RIP-1, RIP-3, and caspase-8, and activating the caspase-3-related pathway [70,71,88]. All three phosphorylated kinases, namely extracellular signal-regulated kinase (ERK), p38 mitogen-activated protein kinase (p38), and c-Jun N-terminal kinase (JNK), exhibited significant upregulation, thereby indicating the activation of the three MAPK signal pathways in response to pristine graphene exposure. This activation subsequently triggered the downstream Bcl-2 protein family, initiating the execution of mitochondrial-related apoptosis. Concurrently, the pro-apoptotic members of the Bcl-2 family including Bim and Bax were additionally activated by the TGF-β/Smads signal pathway, leading to the induction of mitochondrial outer membrane permeabilization and the relocation of mitochondrial pro-apoptotic factors to the cytosol, which ultimately triggered the initiation of the apoptosis-related caspase cascade [87].

## 6. Conclusions and Perspectives

Due to their unique physicochemical properties, GBNs are extensively utilized in various fields such as high-performance wires, sensors, batteries, bio-detection, and drug delivery. Numerous in vivo and in vitro experimental investigations have consistently demonstrated that GBNs elicit acute and chronic inflammation, predominantly characterized by neutrophils, along with edema, granulomas, fibrosis, and other detrimental impacts on the respiratory system. In particular, pristine graphene, rGO, and some specific functionalized GBNs may have less toxic effects in the lungs. In brief, the toxicity of GBNs mainly depends on their specific type, hydrophobicity, lateral size, dosage and duration of exposure, surface chemistry and size, as well as other pertinent factors. 

The currently identified toxicology mechanisms of GBNs mainly focus on oxidative stress, inflammation, genotoxicity, epigenetics, and apoptosis in in vitro cellular assays. However, there is a lack of population epidemiological evidence and in-depth animal experiments regarding the toxic effects of GBNs on human beings and their underlying mechanisms of toxicity in vivo. Consequently, future investigations should emphasize the following aspects for a thorough and comprehensive exploration. The toxic effects of different physicochemical properties of GBNs can be assessed by in vitro and in vivo experiments in combination with scanning electron microscopy and transmission electron microscopy, with particular emphasis on elucidating the impacts of lay number, lateral size, and surface modification on the interactions or mechanisms between GBNs and genes, DNA, proteins, metabolism, and signal pathways, as well as organization of the cytoskeleton, mitosis, and organelle integrity. In addition to the potential mechanism of GBNs in diminishing macrophage-mediated defenses and clearance in the lungs, the interaction of the mucus barrier in the respiratory tracts and the lung lining fluid in the alveoli with GBNs should be comprehensively explored. These endeavors would contribute to the assessment of the biological safety of GBNs and facilitate the identification of the least hazardous GBNs, thereby developing novel graphene-based nanomedicine and fostering advancements in environmental and biomedical health. Furthermore, it is also imperative to establish methods and standards for monitoring GBNs in occupational and environmental settings and to conduct epidemiological surveys of populations exposed to GBNs in workplace and living environments, with the aim of analyzing sources, routes of exposure, internal and external exposure levels, and health effects. These studies will serve as the foundation for establishing exposure thresholds and conducting health risk assessments for GBNs.

## Data Availability

The data used in this work can be obtained in the corresponding literatures.
